# Tobacco Control: Visualisation of Research Activity Using Density-Equalizing Mapping and Scientometric Benchmarking Procedures

**DOI:** 10.3390/ijerph6061856

**Published:** 2009-06-12

**Authors:** Bianca Kusma, Cristian Scutaru, David Quarcoo, Tobias Welte, Tanja C. Fischer, Beatrix Groneberg-Kloft

**Affiliations:** 1 Department of Information Science, Institute of Occupational Medicine, Charité-Universitätsmedizin Berlin, Free University Berlin and Humboldt-University Berlin, Berlin, Germany; E-Mails: bianca.kusma@charite.de (B.K.); david.quarcoo@charite.de (D.Q.); 2 Department of Respiratory Medicine, Hannover Medical School, Hannover, Germany; E-Mail: welte.tobias@mh-hannover.de; 3 Allergy-Centre-Charité, Department of Dermatology and Allergy, Charité-Universitätsmedizin Berlin, Free University Berlin and Humboldt-University Berlin, Berlin, Germany; E-Mail: tanja.fischer@charite.de; 4 Otto-Heubner-Centre, Charité-Universitätsmedizin Berlin, Free University Berlin and Humboldt-University Berlin, Berlin, Germany; E-Mail: beatrix.groneberg-kloft@charite.de

**Keywords:** tobacco control, scientometrics, density equalizing mapping

## Abstract

**Background:**

Tobacco smoking continues to be a major preventable cause of death and disease and therefore tobacco control research is extremely important. However, research in this area is often hampered by a lack in funding and there is a need for scientometric techniques to display research efforts.

**Methods:**

The present study combines classical bibliometric tools with novel scientometric and visualizing techniques in order to analyse and categorise research in the field of tobacco control.

**Results:**

All studies related to tobacco control and listed in the ISI database since 1900 were identified by the use of defined search terms. Using bibliometric approaches, a continuous increase in qualitative markers such as collaboration numbers or citations were found for tobacco control research. The combination with density equalizing mapping revealed a distinct global pattern of research productivity and citation activity. Radar chart techniques were used to visualize bi- and multilateral research cooperation and institutional cooperation.

**Conclusions:**

The present study supplies a first scientometric approach that visualises research activity in the field of tobacco control. It provides data that can be used for funding policy and the identification of research clusters.

## 1. Introduction

The 20th century has witnessed the birth and development of a dangerous new epidemic: in the last 50 years, due to the constant rise in tobacco consumption, tobacco smoke has become an important health hazard in numerous countries [1,2]. In this respect it has become clear that nicotine is the leading substance concerning the development of the addiction [3]. Nicotine itself is known to be a powerful poison – similar to prussic acid. Nevertheless, most health-related consequences are attributable to the 2,500 toxins in the tobacco plant and to the some 4,000 substances present in tobacco smoke [4]. In the U.S.A. and many E.U. countries, tobacco causes more deaths than any other dependence - producing substance. The developing countries will imitate this trend if they continue to increase their tobacco consumption. It is estimated that tobacco causes about five million deaths per year worldwide [5]. Therefore, tobacco control issues are extremely important. The WHO’s 2004 Framework Convention on Tobacco Control (FCTC), signed by 168 nations, seeks to reverse the adverse health effects of tobacco. Tobacco control measures may include numerous procedures such as higher tobacco taxes, smoke free public areas, tobacco content regulation, tobacco warning labels, anti-tobacco education efforts, restrictions on tobacco advertising, sponsorships, and promotions, tobacco cessation, and anti-smuggling provisions. In summary, these measures may help to reduce the burden of disease which is caused by tobacco smoke [6–8]. However, precise scientometric approaches have not been implemented so far to analyse previous research activities in the specific field of tobacco control. Therefore the present study analysed scientometric parameters in the field of tobacco control using classical bibliometric techniques in combination with novel visualizing calculations and large databases. In specific, we aimed to assess the following parameters: 1) Total number of published items and citations, average references and average authorship numbers, country total numbers of published items, country average citation index, country research network parameters (numbers of bilateral country cooperation) and institutional network parameters (numbers of bilateral institutional cooperation).

## 2. Results and Discussion

### 2.1. Total Number of Published Items and Citations, Average References and Average Authorship

**Figure 1.**
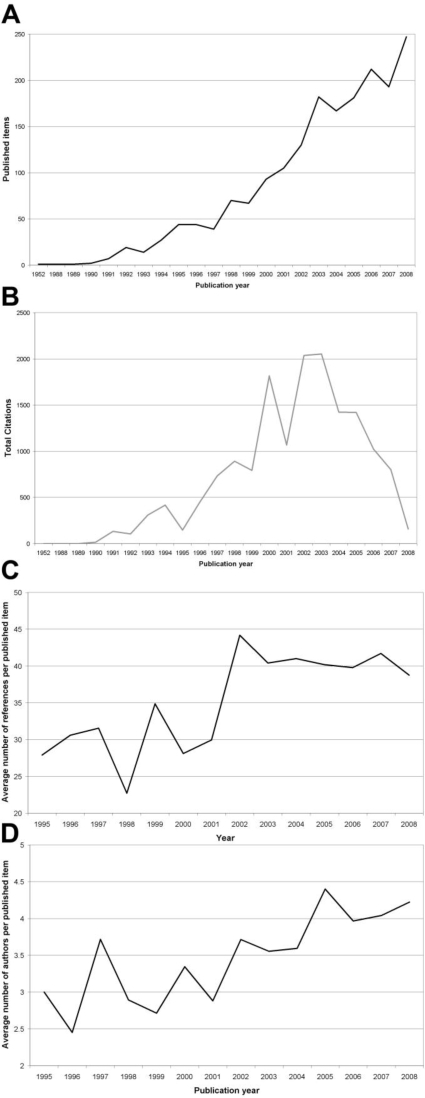
(A) Analysis of total number of published items. (B) Analysis of total number of citations. (C) Average number of references per published item per year. (D) Average number of authors per published item per year.

The number of published items was used as an index of quantity of research productivity. In total, a number of 1,846 “tobacco control” related articles were identified in the Web of Science database. The first article was published in 1952. The year 2008 holds the largest number of published items (247), followed by 2006 (212) and 2007 (193) (Figure 1A). Concerning total number of citations, the articles published in the year 2003 lead with a total number of 2,052 citations followed by 2002 (2,036), 2000 (1,817) and 2004 (1,423, Figure 1B). When analyzing the average number of references per published item per year since 1995, values ranged between 22.71 references per article in the year 1998 and 44.17 in the year 2002 (Figure 1C). Concerning the average number of authors per article between 1995 and 2008, a range was found between 2.45 in the year 1996 and 4.4 authors per article in the year 2005 (Figure 1D).

### 2.2. Country Research Analysis

**Figure 2.**
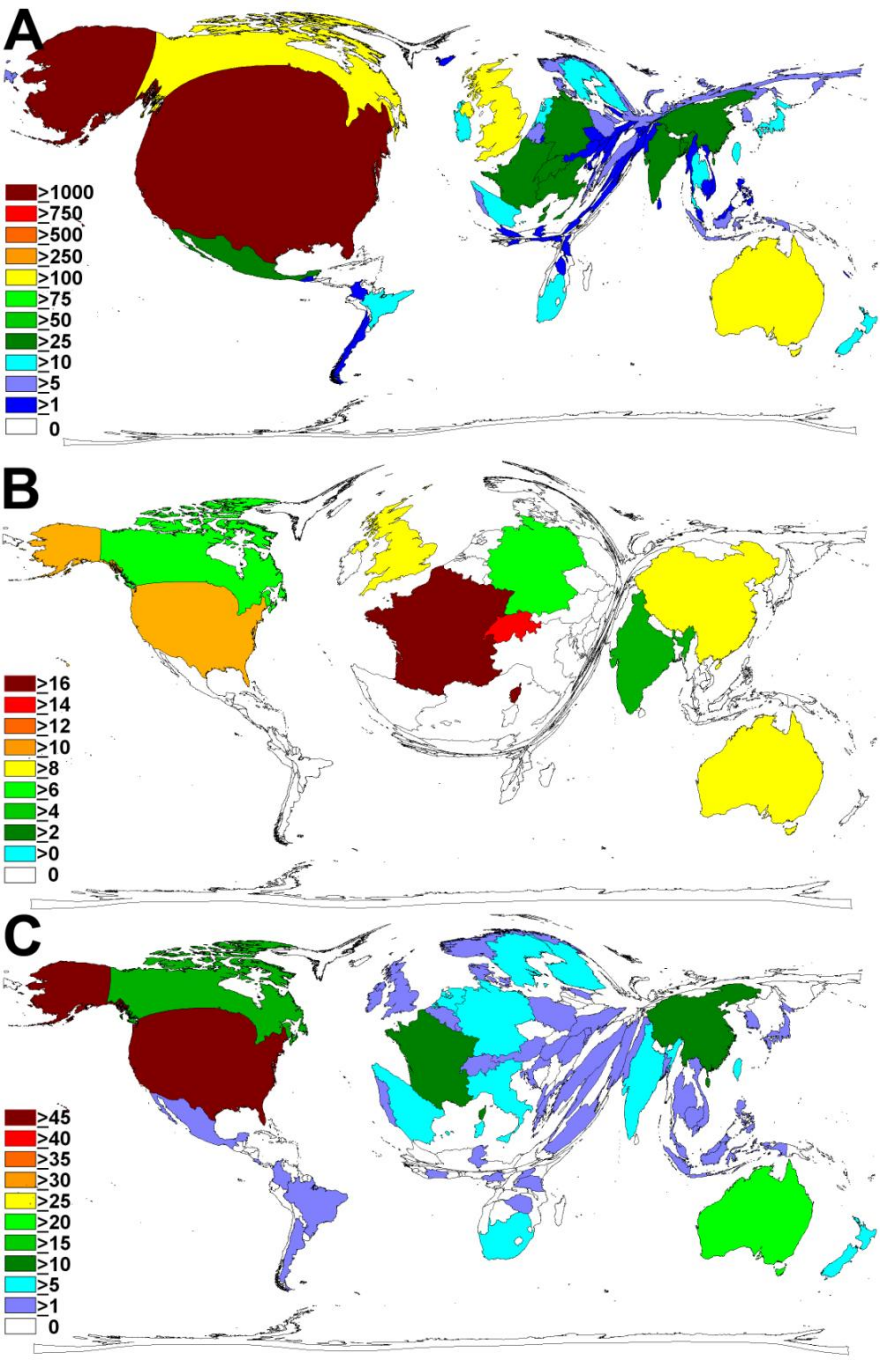
Density-equalizing calculations. (A) Map illustrating the number of contributions for each country for tobacco control related articles for the period 1900 – 2008. (B) Map illustrating the average citations per published item for each country for tobacco control related articles for the period 1900 – 2008. (C) Map illustrating the country-modified h-index in the period 1900 – 2008. In all maps, the area of each country was scaled in proportion to the respective parameter. Colours encode the total number of contributions (A), the average citations per published item (B), and the country-modified h-index (C).

The United States is the country with the highest output of “tobacco control” articles, with a total of 1,078 articles. Australia is in second place with 178, followed by the United Kingdom with 157, Canada with 156, China with 48, Switzerland with 46 and Germany with 35 articles. Density-equalizing mapping was used to illustrate the research output by territorial resizing (Figure 2A). In this visualisation, the US clearly dominates the cartogram.

When average citations per published item related to “tobacco control” were analysed, articles originating from France lead with 16.41 average citations, followed by Swiss articles with 14.26, U.S. articles with 10.67, Chinese articles with 9.31, U.K. articles with 8.96 and Australian articles with 8.59. Density equalizing mapping illustrates these results (Figure 2B) which differ from the total number of published items cartography (Figure 2A).

In the analysis of the country-modified h-index the United States lead with a value of 45, followed by Australia with a value of 20, Canada 17, France 13 and China 10, respectively. These results are also presented in form of a cartogram (Figure 2C).

### 2.3. Country Research Network Analysis

**Figure 3.**
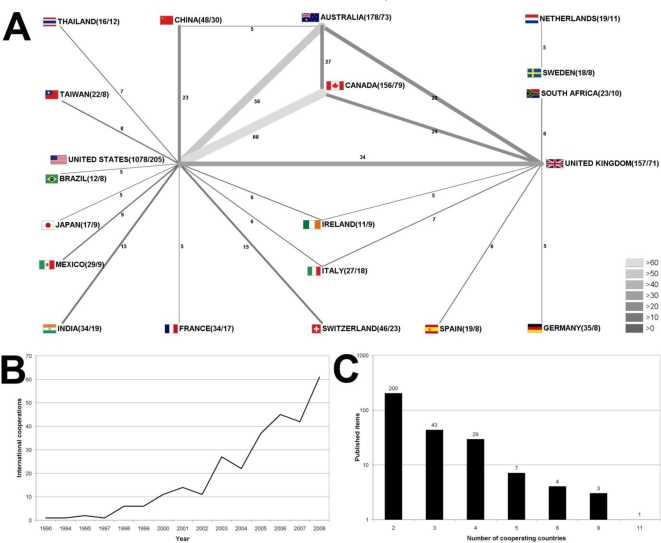
Country network analysis. (A) Radar chart visualising bilateral networking between countries for the overall number of cooperation between the two countries. Greyscale and size of bars encode the number of bilateral cooperation. (B) Evolution of international cooperation over since 1990. (C) Total numbers of published items with authors originating from two, three or more countries (bi-, tri, and multilateral cooperation.

There is an overall increase in international research cooperation present. In 1990, the first international cooperation article was published. 2008 represents the year with the largest number of cooperation articles (61), followed by 2006 (45) and 2007 (42) (Figure 3B). To visualise research networking for “tobacco control” articles, the radar chart technique was used and it was found that with a number of 68 bilateral cooperation articles, Canada and the United States are the leading cooperating countries. This is followed by the cooperation between Australia and the United States (56) and the United Kingdom and the United States (34) (Figure 3A). The largest number of cooperation articles is the result of a bilateral cooperation between two countries (200). Forty three articles are the result of trilateral collaborations and 29 articles originate from the collaboration between four countries (Figure 3C).

### 2.4. Institutional Research Network Analysis

**Figure 4.**
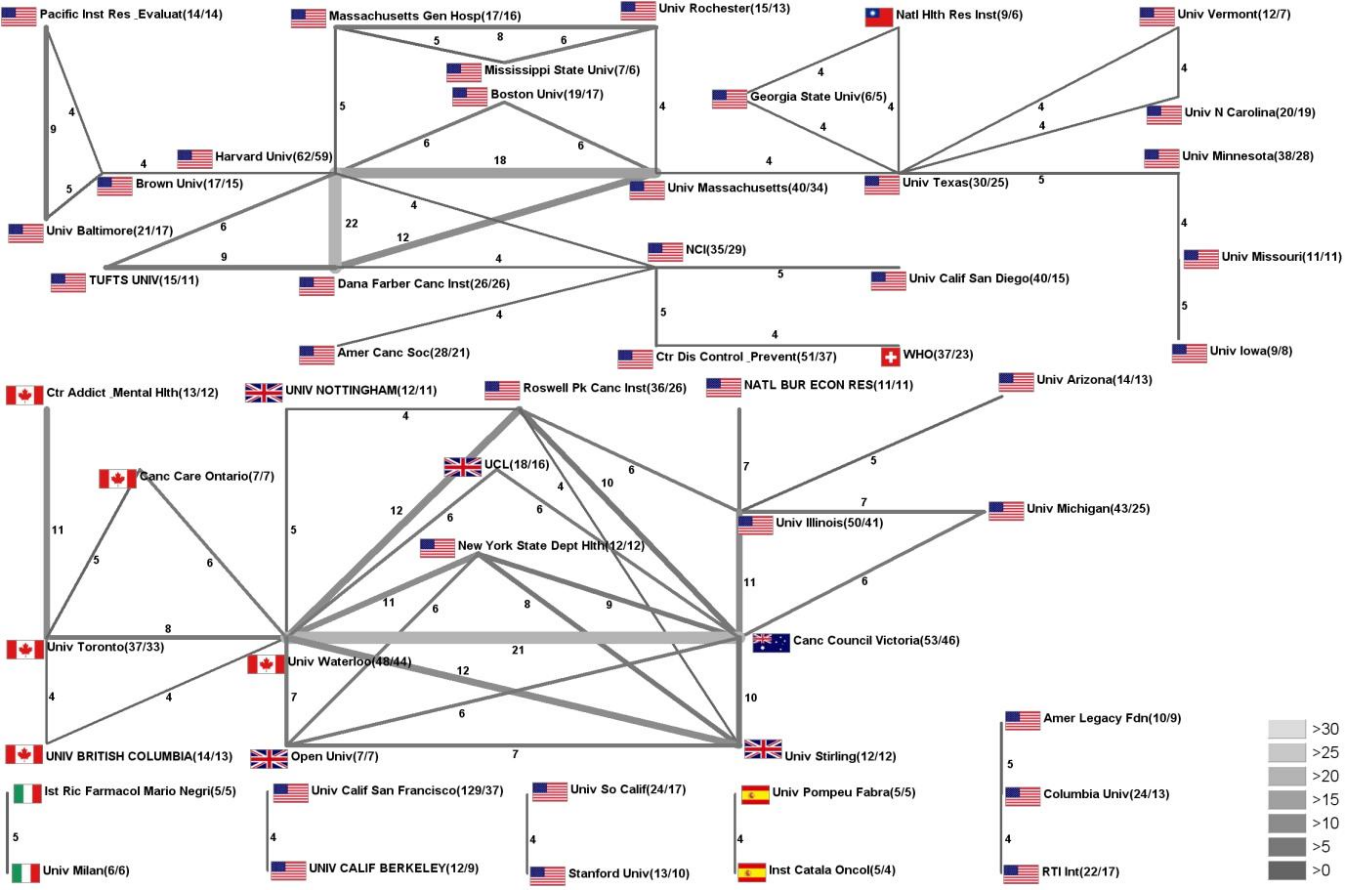
Institutional research network analysis. Radar chart visualising networking between different institutions. Greyscale and size of bars encode the number of cooperation.

In order to identify and visualise leading institutional networks the radar chart technique was applied (Figure 4) and demonstrated interesting findings: The highest cooperation numbers were present for cooperation between the institutions “Harvard Univ.” and “Dana Farber Canc Inst.”, with a number of 22 articles. In second place cooperation between “Univ. Waterloo” and “Canc. Council Victoria” were found (21). The third highest cooperation number was present for “Dana Farber Canc. Inst.” and “Univ. Massachusetts”, with 18 published items.

### 2.5. Subject Area Analysis

**Figure 5.**
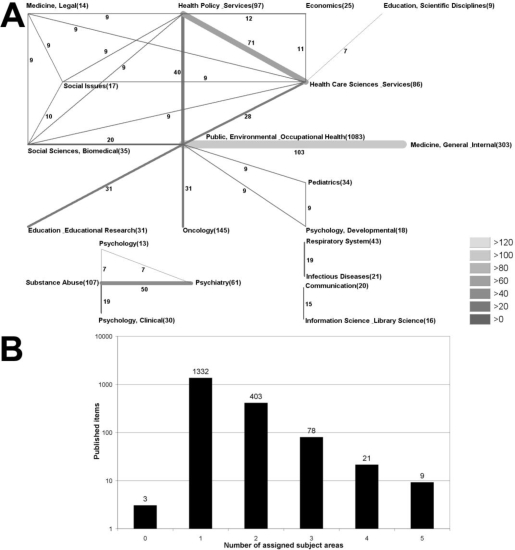
Subject area analysis. (A) Radar chart visualising the combination of subject field denominations of published items with more than two subject fields. Greyscale and size of bars encode the number of assigned articles. (B) Total number of assigned subject areas.

The great majority of the analysed articles were assigned to only one subject area (1,332), followed by 403 articles which were assigned to two subject areas and 78 for three subject areas (Figure 5b). The analysis of the combination of these subject areas were visualized in a radar chart (Figure 5a): The most frequently found combination is present between the subject areas “Public, Environmental & Occupational Health” and “Medicine, General & Internal” (103). The second most frequent combination with a total of 71 articles combines the subject areas “Health Policy & Services” and “Health Care Sciences & Services”. In third place with a total of 50 articles, the combination “Substance Abuse” and “Psychiatry” was found.

### 2.6. Journal Analysis

**Figure 6.**
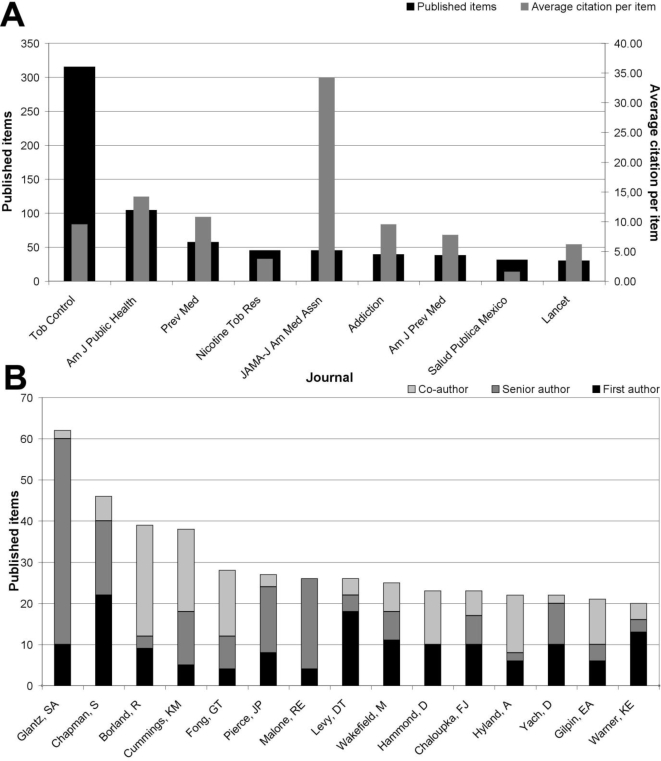
Journal and author analysis. A: Ranking of most productive journals. B: Ranking of most productive authors.

The highest number of “tobacco control”-related articles were published in the journal “*Tobacco Control*” (315), followed by the “*American Journal of Public Health*” (104), “*Preventive Medicine*” (57), “*Nicotine and Tobacco Research*” (45) and the “*JAMA – Journal of the American Medical Association*” (45) (Figure 6A). Concerning the average citation per item, the journal “*JAMA – Journal of the American Medical Association*” holds the highest rate with a value of 34.24 citations per published item, followed by the “American Journal of Public Health” (14.16) and “Preventive Medicine” (10.74) (Figure 6B).

### 2.7. Author Analysis

In a last step, an author analysis was performed for the 1,095 “tobacco control”- related articles. The author Glantz, S.A. was found to be the author/co-author with the highest number of articles related to “tobacco control” (62), followed by Chapman, S. (46), Borland, R. (39) and Cummings, K.M. (38) (Figure 6B). Glantz, S.A. also displayed the highest number of senior authorships (50), followed by Malone, R.E. (22) and Chapman, S. (18). Chapman, S. wrote the highest number of articles as first author (22), followed by Levy, D.T. (18) and Wakefield, M. (11) (Figure 6B).

### 2.8. Discussion

Among other environmental [9–11] and occupational [12–14] airborne hazards, tobacco smokes exerts an enormous toll on the global burden of disease. Consequently, research in the area of tobacco control is urgently needed. While previous bibliometric studies have assessed research in the field of tobacco in general [15,16], the present study focuses on the area of tobacco control using a combination of novel visualizing tools such as density equalizing mapping and classic bibliometric tools such as publication and citation analysis.

Smith *et al*. recently addressed the issue of bibliometric research efforts in the area of environmental and occupational health [17]. It was demonstrated that bibliometrics - defined as the use of mathematical techniques to investigate publishing and communication patterns in the distribution of information – has been an established approach in occupational and industrial health for about 20 years [18]. In this respect, McCunney and Harzbecker published a study on citation patterns in 1992 [19]. New proposals for improved measures of quality indices were published by a number of scientists [20], Garfield [21] and Gehanno and Thirion [22]. However, in the specific field of tobacco control, no large scaled analysis was performed between the years 1900 and 2008 and previous bibliometric studies focused on tobacco more in general [15,16]. We therefore used novel scientometric techniques in combination with visualizing techniques and were able to demonstrate an increasing number of international networks. This need to be interpreted in the context of tobacco control research and funding: Tobacco-related diseases are estimated to exert a major burden of disease, with most of the costs due to both diagnosis and therapy. Therefore, numerous national and international tobacco control research networks were founded by governmental and non-governmental institutions. These research networks are at least partly responsible for the increasing number of multilateral cooperation which have been found for tobacco control related publications in the present study.

For the present study, it is important to realise that the analysis of tobacco control related articles cannot be regarded as completely representative of global occupational research activity in this field. In this respect, a bias is represented by the database (Web of Science) which was used. Whereas this database in among the largest global biomedical databases, there are still publications which cannot be traced via this system. However, it can be hypothesized that the presently observed trends represent the ongoing efforts in this field. In general, the U.S. led research efforts. This was also evident after visualisation by the density equalizing mapping technique using Gastner and Newman’s algorithm [23]. This technique also illustrates that in other indices such as the average citation per country parameter, other countries such as France take a leading position.

Whereas the number of published items was currently considered as an index of quantity of research productivity – lead by the U.S., citation analysis may be used as an indicator for research quality. However, quality indicators need to be regarded critically and therefore, the data should not be over interpreted as indicated by numerous previous articles [20–22].

## 3. Experimental Section

### 3.1. Data Source and Time Span

Data was retrieved from the database Web of Science Thomson Reuters [24,25]. The period 1900 to 2008 was used as restriction for the publication date since the data entry for 2009 has not been terminated so yet.

### 3.2. Search Strategies

All published items including the search term “tobacco control” (in the field “topic”) were selected and downloaded for analyzing purposes using a web interface (last update: May 5, 2009). No additional filters were used. As statistical and graphical software tools, SPSS 17.0 and 3Dfield were used.

### 3.3. “Quality” Parameter Analysis

For countries with at least 30 published items on “tobacco control”, the average citation per published article was calculated. The original h-index for authors defined by Hirsch [26], is quality index (h), where h is the maximum number from the total number of articles written by a given author where each one of these h articles have been at least h times cited. It is useful to assess both productivity and quality of scientific productivity. This h-index was modified and extrapolated to the articles originating from a specific country. In this respect, a country-specific modified h-index was calculated in order to assess the “quality” of articles from a specific country.

### 3.4. Density-Equalizing Mapping

Density-equalizing mapping procedures were used as described in previous studies [27,28]. In brief, all territories were resized according to 1) the number of published items related to “tobacco control”, 2) the average citations and 3) the modified country h-index, respectively [29]. For the resizing procedure the area of each country was scaled in proportion to the variables. The calculations of the procedure are based on Gastner and Newman’s algorithm [23].

### 3.5. Analysis of Bilateral Country and Institution Cooperation

The analysis of bilateral country and institution cooperation was chosen to assess research networks. A bilateral cooperation between two countries was defined when at minimum one author originates from one country and at least one other author from a second country. A matrix with all identified countries was computed and filled with the appropriate values for the cooperation for each pair of countries. A second software module was developed to interpret the matrix and transform the figures into vectors. The thickness of the vector quantified the number of cooperation articles between the two countries. A threshold of at least five cooperation was set for the multilateral country analysis and a threshold of at least four cooperation was set for the multilateral institution cooperation analysis in order to improve the readability. The results were visualised graphically using the radar chart technique.

### 3.6. Analysis of Subject Categories

Subject category analysis was performed in order to identify the subjects that have the strongest link to tobacco control. The radar chart technique was used to illustrate the number of published items that are denominated by at least two subject categories.

### 3.7. Journal and Author Analysis

In order to identify the journals and authors with the highest publication activities in the field of tobacco control, the set of published items was analysed for journal origin and contributing authors. The journals were also monitored for citations and the authors for first-, senior- or co-authorships.

## 4. Conclusions

The present study represents a large scale scientometric Web of Science database analysis and visualisation of “tobacco control” publications using density equalizing calculations and chart techniques. The proposed aims of the studies were to analyse total numbers of published items and citations, average references and average authorship numbers, country total numbers of published items, country average citation index, country research network parameters and institutional network parameters. It can be concluded that the field has witnessed a strong increase in research productivity and networking over the past years. The techniques established here can be of use for future bibliometric studies in this field.
